# Aspergillus fumigatus Bloodstream Infection in the Absence of Classic Risk Factors: Expanding the Spectrum of Invasive Aspergillosis

**DOI:** 10.7759/cureus.80576

**Published:** 2025-03-14

**Authors:** Marcos Molina, Nismat Javed, Shalini Penikilapate, Oladipo Alao

**Affiliations:** 1 Internal Medicine, BronxCare Health System, New York, USA; 2 Medicine, BronxCare Health System, New York, USA

**Keywords:** aspergillus fumigatus, fungal bloodstream infection, immunocompromised host, invasive aspergillosis, non-neutropenic aspergillosis, unusual presentations of aspergillosis

## Abstract

We report an unusual case of *Aspergillus fumigatus* fungemia in a 65-year-old male with multiple comorbidities including, human immunodeficiency virus (HIV), chronic obstructive pulmonary disease (COPD), diabetes mellitus, hepatitis C, and metastatic small cell lung cancer on chemotherapy. He presented with pneumonia and acute hypoxic respiratory failure requiring intubation. The patient developed septic shock and a peripherally inserted central catheter (PICC-line)-associated methicillin-sensitive *Staphylococcus aureus* (MSSA) bacteremia treated with intravenous (IV) cefazolin. Blood cultures unexpectedly revealed *A. fumigatus* despite a lack of classic risk factors, other than advanced acquired immune deficiency syndrome (HIV/AIDS), such as prolonged neutropenia or stem cell transplantation. Suspected sources included disseminated infection from the lungs and/or the infected PICC line. This case highlights the diagnostic and therapeutic challenges of invasive aspergillosis, a rarely reported and poorly understood entity with a high mortality rate. Further studies are needed to better characterize the epidemiology, risk factors, and optimal management of *Aspergillus *fungemia in diverse immunocompromised populations.

## Introduction

This report describes an unusual case of *Aspergillus fumigatus* fungemia in a 65-year-old male with multiple comorbidities including human immunodeficiency virus (HIV), asthma, chronic obstructive pulmonary disease (COPD), diabetes, hepatitis C, prior history of tuberculosis (TB), substance abuse history, and recently diagnosed metastatic small cell lung cancer. *Aspergillus *species are ubiquitous environmental molds that most commonly cause invasive pulmonary infections in immunocompromised hosts [1]. In contrast, *Aspergillus *fungemia is a rare entity, even among high-risk patients, with an incidence of less than 1% in hematopoietic stem cell transplant recipients and cancer patients [2,3]. The patient was admitted to the intensive care unit (ICU) with pneumonia and acute hypoxic respiratory failure requiring intubation. He developed septic shock and a peripherally inserted central catheter (PICC-line)-associated methicillin-sensitive *Staphylococcus aureus* (MSSA) bacteremia, treated with intravenous (IV) cefazolin. Blood cultures also unexpectedly grew *A. fumigatus*, which is rare and distinct from the more common invasive pulmonary aspergillosis [4].

## Case presentation

A 65-year-old male with a past medical history significant for HIV (CD4 count 163 cells/mm^3^), asthma, COPD, diabetes mellitus, hepatitis C, prior TB, and ongoing substance abuse was admitted for acute hypoxic respiratory failure secondary to pneumonia and COPD exacerbation. He was found to have metastatic small cell lung cancer diagnosed via transthoracic lung biopsy and completed two cycles of chemotherapy with cisplatin and etoposide.

The patient subsequently developed septic shock requiring intubation, vasopressors, and broad-spectrum antibiotics. His hospital course was further complicated by superior vena cava (SVC) syndrome and a left upper extremity deep vein thrombosis, for which he was treated with therapeutic enoxaparin.

On hospital day 52, the patient developed recurrent fevers and was transferred back to the ICU for management of septic shock. Blood cultures grew MSSA, and a transesophageal echocardiogram was negative for endocarditis. He was started on IV cefazolin for a PICC-line-associated MSSA bacteremia. Repeat blood cultures on hospital day 54 eventually grew *A. fumigatus* (which resulted after the patient’s demise) in addition to MSSA.

The infectious disease team was consulted and recommended continuing IV cefazolin for the MSSA bacteremia and adding caspofungin for fungal coverage. Atovaquone was continued for pneumocystis jirovecii prophylaxis. Tenofovir was discontinued due to concern for contributing to his acute kidney injury, and his antiretroviral regimen was changed to dolutegravir/rilpivirine plus emtricitabine pending further evaluation of his hepatitis B status.

**Figure 1:**
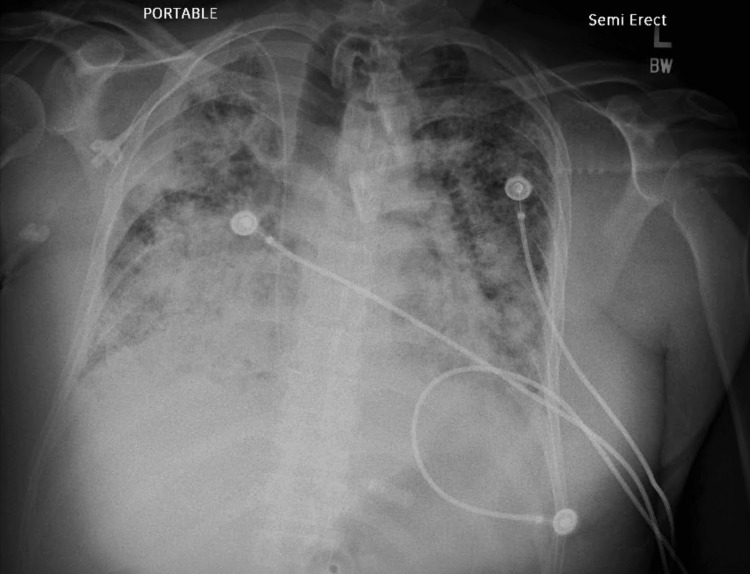
Patient’s X-ray at day 61

Despite these interventions, the patient remained ventilator-dependent and in septic shock on multiple vasopressors. His white blood cell count climbed from 15.1 k/uL to 30.9 k/uL over the next several days. Chest radiography revealed persistent bilateral airspace opacities (Figure 1). The patient ultimately died of his illness on hospital day 62.

## Discussion

The patient in this case had several comorbidities that likely increased his risk for invasive fungal infections, including diabetes, steroid use, and metastatic lung cancer while undergoing chemotherapy. He also had acquired immune deficiency syndrome (AIDS) with a CD4 count of 163 cells/mm^3^, while indicating significant immunosuppression, which was not as low as typically seen in HIV patients with invasive aspergillosis [5]. However, he lacked many of the classic risk factors associated with *Aspergillus *fungemia, such as profound neutropenia, hematologic malignancy, or stem cell transplantation [6,7]. Table 1 presents the significant nuances observed across several case reports of *Aspergillus *fungemia.

**Table 1:** Comparison of several case reports y/o: year-old; AML: acute myelogenous leukemia; GM: galactomannan; CT Scan: computed tomography scan; AIDS: acquired immune deficiency syndrome; COPD: chronic obstructive pulmonary disease; TB: tuberculosis; *A. fumigatus*: *Aspergillus fumigatus*

Author (year)	Demographics	Symptoms	Remarkable labs	Management	Outcome
Batista et al. (2011) [8]	56 y/o M, AML, neutropenia post-chemotherapy	Fever, dyspnea, cough	GM index > 6.0, CT scan: cavitary lung lesions	Voriconazole	Died
Torres et al. (2003) [9]	42 y/o M, AIDS, CD4 < 50 cells/μL	Fever, cough, weight loss	Blood culture: *A. fumigatus*	Itraconazole, amphotericin B	Died
Schweer et al. (2014) [10]	62 y/o M, COPD, TB history	Hemoptysis, dyspnea	CT scan: cavitary lesion, fungal culture:* A. fumigatus*	Voriconazole, bronchial artery embolization	Survived
Garcia-Vidal et al. (2011) [11]	62 y/o M, COPD, corticosteroids	Fever, cough, chest pain	Blood culture: *A. fumigatus*, GM positive	Voriconazole	Survived
Malani and Kauffman (2007) [12]	52 y/o M, AML, neutropenia	Fever, chest pain	Blood culture: *A. fumigatus*	Amphotericin B, voriconazole	Died

The presumed primary source of infection in this case was the lung, given the patient's history of pneumonia. However, dissemination of *Aspergillus *from the lung to the bloodstream is uncommon, estimated to occur in only 2-10% of cases of invasive pulmonary aspergillosis [13]. Moreover, the patient's imaging did not demonstrate the classic "halo sign" or cavitary lesions that are often seen in angio-invasive pulmonary aspergillosis [14]. Table 2 shows the patient's chest X-ray revealed bilateral airspace opacities suggestive of multifocal pneumonia or pulmonary edema but lacked the nodular lesions with surrounding ground-glass halos that are considered characteristic of early invasive aspergillosis [15]. The absence of these typical radiographic findings, as displayed in Figure 1, does not exclude the diagnosis, but it does make it less likely based on imaging alone. Further evaluation with computed tomography (CT) imaging could potentially identify lesions not apparent on plain radiographs.

**Table 2:** Comparative X-ray findings

Finding	Patient's X-ray	Classic invasive pulmonary aspergillosis X-ray
Nodules	No distinct nodules visible	Single or multiple nodules, 1-3 cm in diameter, often with surrounding halo sign [15]
Halo sign	No halo sign present	Halo of ground glass attenuation surrounding a nodule, indicating hemorrhage; seen in early angio-invasive disease [14]
Cavitation	No clear cavitary lesions	Nodules may cavitate as disease progresses [15]
Consolidation and infiltrates	Bilateral airspace opacities consistent with multifocal pneumonia or edema	Consolidation and ground-glass infiltrates can be present, but typically localized around nodular lesions [15]
Pleural effusions	No significant pleural effusions	Uncommon finding, unless related to other complications [15]

Another notable aspect of this case was the presence of a PICC line, which may have served as a nidus for fungal infection. Intravascular catheters have been identified as a risk factor for *Aspergillus *fungemia in several studies [6,16]. However, concomitant bacteremia with MSSA made it difficult to determine the significance of the positive fungal blood cultures. It is possible that the *Aspergillus *represented a transient or contaminated sample rather than a true infection.

The optimal treatment of *Aspergillus *fungemia typically involves a combination of antifungal therapy with an azole or amphotericin B and the removal of any infected vascular catheters [17]. In this case, the PICC line was appropriately removed, and caspofungin was empirically started for suspected candidemia. However, *Aspergillus *fungemia is associated with high mortality rates, approaching 90% in some series [18].

## Conclusions

In conclusion, this case illustrates an atypical presentation of *Aspergillus *fungemia in a patient with multiple comorbidities but lacking classic risk factors such as neutropenia or stem cell transplantation. The fungemia may have originated from an infected PICC line in the setting of underlying pneumonia. The case highlights the challenges in diagnosing and managing this rare but serious condition, particularly in the context of concomitant bacterial infection and critical illness. Further research is needed to better understand the epidemiology, risk factors, and optimal empiric treatment strategies for *Aspergillus *fungemia in diverse patient populations.

## References

[1] Kousha M, Tadi R, Soubani AO (2011). Pulmonary aspergillosis: a clinical review. Eur Respir Rev.

[2] Kontoyiannis DP, Marr KA, Park BJ (2010). Prospective surveillance for invasive fungal infections in hematopoietic stem cell transplant recipients, 2001-2006: overview of the Transplant-Associated Infection Surveillance Network (TRANSNET) Database. Clin Infect Dis.

[3] Montagna MT, Lovero G, Coretti C (2014). SIMIFF study: Italian fungal registry of mold infections in hematological and non-hematological patients. Infection.

[4] Denning DW (1998). Invasive aspergillosis. Clin Infect Dis.

[5] Holding KJ, Dworkin MS, Wan PC, Hanson DL, Klevens RM, Jones JL, Sullivan PS (2000). Aspergillosis among people infected with human immunodeficiency virus: incidence and survival. Adult and adolescent spectrum of HIV disease project. Clin Infect Dis.

[6] Parody R, Martino R, Sánchez F, Subirá M, Hidalgo A, Sierra J (2009). Predicting survival in adults with invasive aspergillosis during therapy for hematological malignancies or after hematopoietic stem cell transplantation: single-center analysis and validation of the Seattle, French, and Strasbourg prognostic indexes. Am J Hematol.

[7] Baddley JW, Andes DR, Marr KA (2010). Factors associated with mortality in transplant patients with invasive aspergillosis. Clin Infect Dis.

[8] Batista MV, Pierrotti LC, Abdala E (2011). Endemic and opportunistic infections in Brazilian solid organ transplant recipients. Trop Med Int Health.

[9] Torres HA, Rivero GA, Lewis RE, Hachem R, Raad II, Kontoyiannis DP (2003). Aspergillosis caused by non-fumigatus Aspergillus species: risk factors and in vitro susceptibility compared with Aspergillus fumigatus. Diagn Microbiol Infect Dis.

[10] Schweer KE, Bangard C, Hekmat K, Cornely OA (2014). Chronic pulmonary aspergillosis. Mycoses.

[11] Garcia-Vidal C, Barba P, Arnan M (2011). Invasive aspergillosis complicating pandemic influenza A (H1N1) infection in severely immunocompromised patients. Clin Infect Dis.

[12] Malani AN, Kauffman CA (2007). Changing epidemiology of rare mould infections. Drugs.

[13] Denning DW (1996). Therapeutic outcome in invasive aspergillosis. Clin Infect Dis.

[14] Greene RE, Schlamm HT, Oestmann JW (2007). Imaging findings in acute invasive pulmonary aspergillosis: clinical significance of the halo sign. Clin Infect Dis.

[15] Kang EY, Kim DH, Woo OH, Choi JA, Oh YW, Kim CH (2002). Pulmonary aspergillosis in immunocompetent hosts without underlying lesions of the lung: radiologic and pathologic findings. AJR Am J Roentgenol.

[16] Cornillet A, Camus C, Nimubona S (2006). Comparison of epidemiological, clinical, and biological features of invasive aspergillosis in neutropenic and nonneutropenic patients: a 6-year survey. Clin Infect Dis.

[17] Walsh TJ, Anaissie EJ, Denning DW (2008). Treatment of aspergillosis: clinical practice guidelines of the Infectious Diseases Society of America. Clin Infect Dis.

[18] Vigouroux S, Morin O, Moreau P (2005). Zygomycosis after prolonged use of voriconazole in immunocompromised patients with hematologic disease: attention required. Clin Infect Dis.

